# Why has the rise in obesity not reversed the decline in cardiovascular mortality? Cardiometabolic death rate trends in Brazil (1980-2011): Por que o aumento da obesidade não reverte o declínio da mortalidade por doença cardiovascular? Tendências de mortalidade por evento cardiometabólico no Brasil (1980-2011)

**DOI:** 10.1590/1516-3180.2014.1331979

**Published:** 2014-11-28

**Authors:** Paulo Andrade Lotufo

**Affiliations:** I MD, PhD. Titular Professor, Discipline of Internal Medicine, Faculdade de Medicina da Universidade de São Paulo (FMUSP), São Paulo, Brazil.

In the November 2000 issue of this journal, the editorial made a prediction that the decreasing trend of cardiovascular death rates in Brazil that had been seen in the 1990s would flatten out, as the first effect of the increasing prevalence of obesity in this country.^1^ The premises for this statement were consistent: as the average body mass index increases, diabetes prevalence rates consequently rise and act directly on the incidence and case-fatality rates of cardiovascular diseases, thus causing the descending trend of cardiovascular deaths to flatten out and then possibly start an upward trend. Fourteen years after this editorial, I regret to inform all our readers that this prophecy (which I wrote) did not come to pass. The reality is that obesity is increasing in Brazil,^2^ as it is everywhere,^3^ and cardiovascular mortality rates are declining in Brazil,^4^ as in most other countries.^5^

To explain this apparent paradox, hypotheses such as the notion that the body mass index is not a good surrogate of adiposity as a risk for atherosclerosis and high blood pressure have been put forward.^6^ Another reason may relate to the improvement of medical care, starting with greater control over risk factors at the primary care level, and especially the introduction of new protocols for cardiovascular emergency and a role for statins/aspirin/beta-blockers after myocardial infarction.^7^ Another possibility is that this paradox may have been caused by a nosological artifact: a shift in the underlying cause of death from cardiovascular diseases to diabetes.

The rationale for considering that there may be a classification bias is that, in most cases, people with diabetes die due to heart disease and stroke. However, this has been changing since 1996, with the adoption of the 10^th^ revision of the International Classification of Diseases (ICD-10), in which both diabetes and a well-defined final cardiovascular event can be considered to be the underlying cause of death. This nosological artifact has been found not to be limited to Brazil and has also been shown in countries like Mexico and the United States.^8^ This rationale has been used recently for comparisons of mortality data between countries.^9^

To test this hypothesis, I analyzed the trends in assignment of the underlying cause of death over a period of three decades from 1980 to 2011, according to gender. This analysis concentrated on the age group from 30 to 69 years, because there are few cardiovascular deaths at younger ages (only 2.5% of cardiovascular deaths were among individuals under 30 years of age during this period), and because a high proportion of such deaths among individuals over the age of 70 years cannot be compared due to comorbidities. Since Brazil adopted ICD-10 only in 1996, mortality data were classified according to the 9^th^ revision (ICD-9) from 1980 to 1995 and thereafter using ICD-10. I analyzed all cardiovascular diseases (chapters VII and IX of ICD-9 and ICD-10, respectively), coronary heart disease (CHD, codes: 410-5 and I-20-25) and stroke (code: 460-8 and I-61-69), separately. Furthermore, I added the cases with diabetes (codes: 205 and E-10-15) to all the cardiovascular diseases, thus creating a category called “cardiometabolic deaths.”

The investigation plan included: (1) review of the numbers in all the categories above, with calculation of the proportion of deaths relating to diagnoses that were listed as ill-defined causes of death; (2) adjustment of all crude death rates using the World Standard Population; (3) calculation of temporal changes to death rates using the Joinpoint 4.1.1 software.^10^

**Figure 1.**
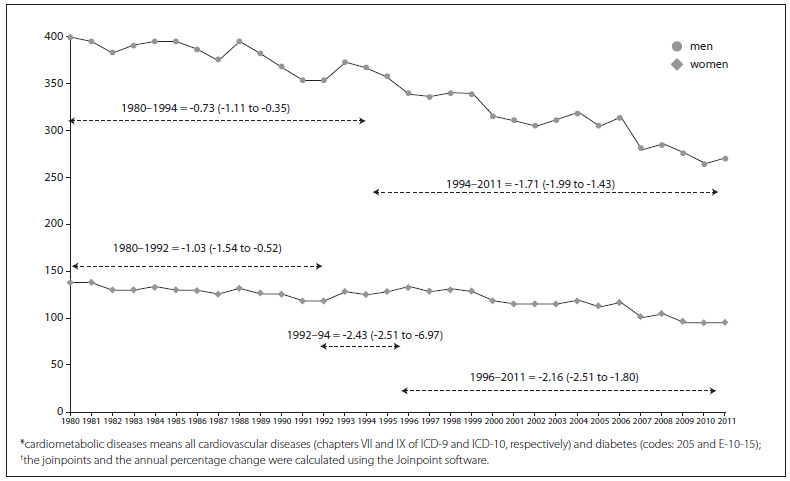
Trends in cardiometabolic disease* death rates in Brazil from 1980 to 2011 with the annual percentage change of each joinpoint^†^ according to sex.

Figure 1 displays the temporal trends of death rates due to cardiovascular diseases and diabetes for men and women in Brazil from 1980 to 2011. The average percentage change for men was different between the periods 1980-94 and 1994-2011, with a faster pace in the second period. For women, a decline in rates was observed from 1980 to 1992; the rates flattened out in 1992-96; and a new downward trend began in 1996. Despite the improvement in coverage of the mortality information system and more reliable quality of death notification, which could have artificially inflated the rates, a persistent decline for cardiometabolic death rates can be seen in all periods. By examining a period with good quality of mortality data, such as 2005 to 2011, it was possible to measure the average annual percentage change in cardiovascular diseases, CHD, stroke and diabetes (Table 1). Significant declines in rates were estimated for all these diseases, except for diabetes in men. Comparison between the sexes showed that only for CHD was the reduction in rates faster for women.

**Table 1.**
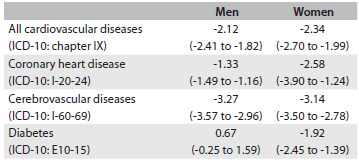
Annual average percentage change (and 95% confidence interval) of age-adjusted death rates in Brazil over the period 2005 to 2011

ICD-10 = International Classification of Diseases, 10^th^ revision.

These temporal changes to death rates in Brazil show that the decline is occurring in the whole country, despite the shifting of nosological causes from cardiovascular diseases to diabetes. The faster decline of stroke death rates, compared with CHD, reveals that the phase of “delayed cardiovascular epidemiological transition” has ended.^11^ Moreover, the agenda for cardiovascular epidemiology needs to deeply consider the meaning of adiposity as a risk factor. Unfortunately, the early studies by Jean Vague in the 1950s,^11^^,^^12^ in which the components of obesity were evaluated separately, were endorsed by epidemiologists only in the early 1980s.^13^ Almost all articles on cardiometabolic trends contain classical final remarks such as “further investigation are needed….” Here, this statement is not a cliché. We need to understand the multiple components of this concept called “obesity.” Finally, I promise to limit my prophecies over the next three years of my term as editor-chief of this journal.
